# Infected femorocele: case report of an extremely rare surgical condition in a middle-aged lady suffering from a painful inguinal swelling

**DOI:** 10.1186/s40792-019-0716-4

**Published:** 2019-10-24

**Authors:** Sabyasachi Bakshi

**Affiliations:** Department of General Surgery, BSMCH, India, Kathghara Lane, Sonatuli, PO and District – Hooghly, Hooghly, West Bengal 712103 India

**Keywords:** Femorocele, Inguinal cyst, Femoral hydrocele, Infected femorocele

## Abstract

**Background:**

The hydrocele of the femoral hernia sac, an extremely rare occurrence, is termed femorocele. Very few authentically reported cases of femorocele are available in the literature. The present case, diagnosed as a case of infected femorocele, was managed successfully by excision of the femorocele sac and repair of the femoral hernia. To the best of the author’s knowledge, it is the first-ever reported case of infected femorocele.

**Case presentation:**

A 30-year-old lady presented with a painful 3 cm × 2 cm swelling in the right inguinal region. Though the swelling was there for 2 years, the pain and indurations started after a trivial blunt trauma over the swelling 7 days ago. The patient was febrile and mild tachycardic but had no dysuria. The oval-shaped, tense-cystic, poorly translucent, non-pulsatile, non-reducible swelling showed no cough impulse. There was also a (1.5 cm × 0.5 cm) palpable right-sided superficial inguinal lymph node. Routine blood and urine analysis reports were normal except leukocytosis (10,000/mm^3^) with neutrophilia. Ultrasonography of the right inguino-labial region revealed a mildly echogenic cystic swelling without any intra-abdominal communication. Exploration of the right inguinal region revealed a cystic (3 cm × 2 cm) swelling, medial to the femoral vessels, containing amber-colored fluid. The distal sac was excised, and anatomical repair of femoral canal defect was done after transfixing the neck of the femorocele sac. Fibro-fatty-collagenous tissue with mixed inflammatory cells along with a flattened mesothelial lining cell layer was found on histopathological examination. Sections from inguinal lymph node showed reactive hyperplasia. Culture of fluid from the sac revealed growth of Escherichia coli. The patient was put on anti-inflammatory drugs and antibiotics according to a sensitivity test. Patient was discharged in stable condition after 5 days. Four months after the operation, the patient is doing well, remaining asymptomatic and without any sign of recurrence.

**Conclusions:**

The hydrocele of the femoral hernia sac is an extremely rare disease. When not infected, it presents a painless inguinal soft cystic swelling, commonly in women of fourth to sixth decade. This was diagnosed intraoperatively in all cases reported till date. Excision of the sac after transfixation of the neck and anatomical repair are the treatment of choice. In elderly patients, with larger defect, the mesh repair can be opted for. The femorocele may also get infected by uropathogens, and proper antibiotics should be used after a sensitivity test.

## Background

Hydrocele, in females, is a rare condition. When found, it commonly involves the canal of Nuck [1]. The hydrocele of the femoral hernia sac, an extremely rare occurrence, is termed femorocele. An extensive search in English medical literature and the Internet revealed less than ten authentically reported cases of femorocele to date. In 1927, H. Bailey first reported a case of ascetic fluid-filled femoral hernia sac [2]. Rives in 1934 published a case report of two femoroceles with no evidence of ascites [3]. McCorkle and Bell reported three cases of femorocele in the University of California Hospital, in 1941 [4]. Mote and Chakravarty in 2009 [5] and Madhivanan et al. in 2016 reported another case of femorocele [6]. Femorocele, when presented in the previously reported cases, was diagnosed provisionally as an irreducible femoral hernia or subcutaneous lipoma. In all reported cases, the diagnosis of femorocele had been made only after surgical exploration. Despite the rarity, surgeons dealing with a femoral hernia should be aware of this clinical entity during the exploration of femoral hernia.

To the best of the author’s knowledge, it is the first-ever reported case of infected femorocele in the world.

## Case presentation

**Fig. 1:**
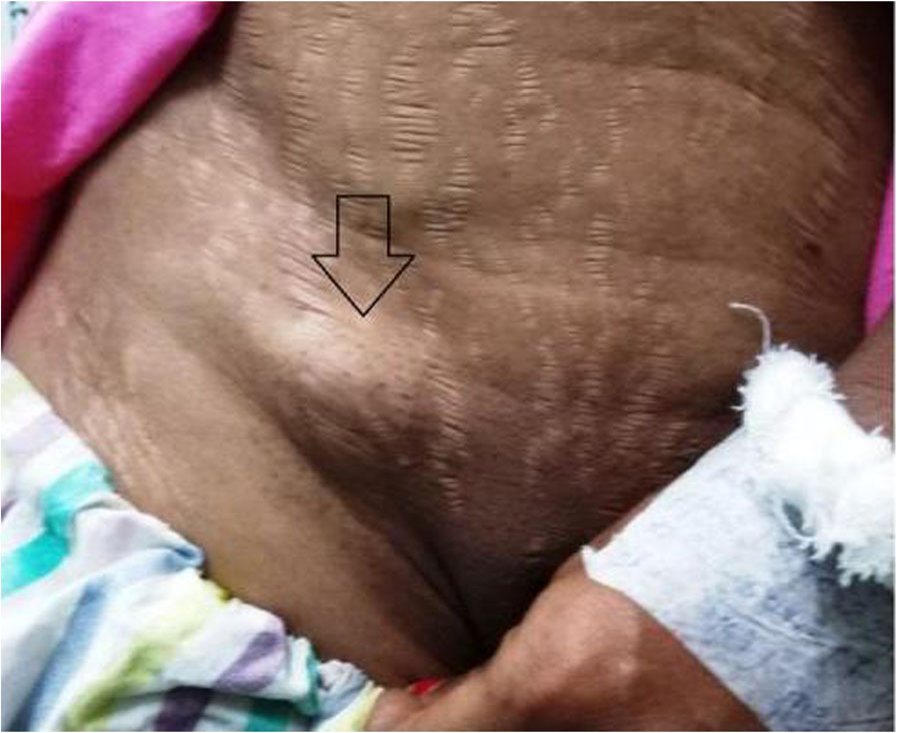
Preoperative image of the right inguinal swelling (below the inguinal ligament and lateral to the pubic tubercle)

A 30-year-old lady, non-smoker with no previous major health problems, presented with fever (101 °F) and a painful swelling in the right inguinal region (Fig. 1) for 7 days after a trivial blunt trauma on the area. The swelling, initially painless and small in size, started 2 years back and gradually attained the present size [3 cm (vertical) × 2 cm (horizontal)] in the last 4–5 months. She developed tenderness and indurations over the swelling after the trauma.

General examination revealed normotensive lady (BMI of 20.5) without any abdominal mass, dysuria, or any sign of intestinal obstruction. She was significant only for the swelling which was located below the inguinal ligament and infero-lateral to the right pubic tubercle. It was oval-shaped, fixed to deeper tissue, tense-cystic, poorly trans-illuminant, non-pulsatile, and irreducible. The swelling was without expansile impulse on coughing and devoid of any bowel sound within it. She also had a single 1.5 cm × 0.5 cm palpable right-sided superficial inguinal lymph node. She neither had a history of any previous abdominal surgery, chronic cough, and coagulopathy nor had she received any treatment for this disease. She had no history of similar illness in her family also.

**Fig. 2:**
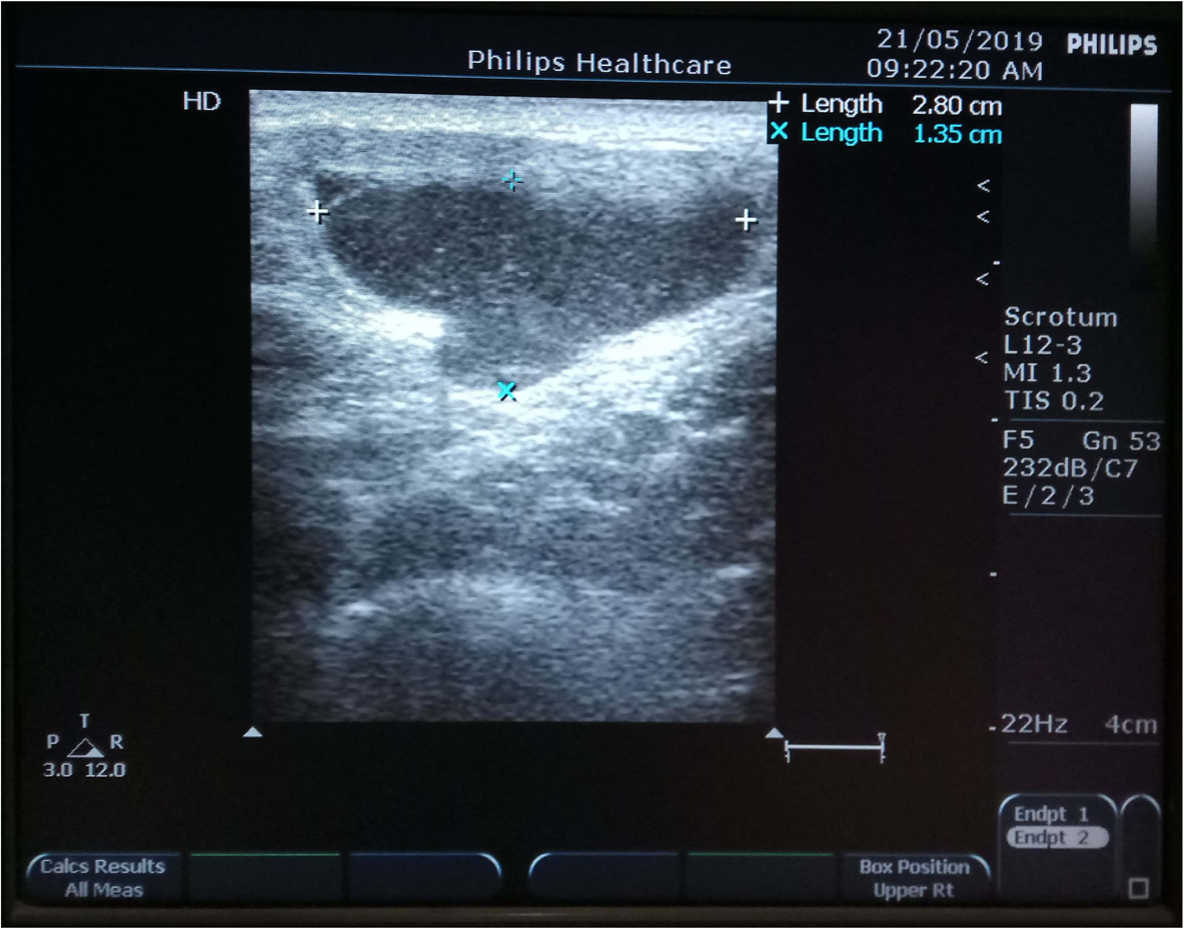
Grayscale ultrasonography of the right inguino-labial region reveals a mildly echogenic cystic swelling of size 2.80 cm × 1.35 cm below the inguinal ligament and medial to the femoral vessels without any intra-abdominal communication

The possibility of incarcerated femoral hernia or infected post-traumatic hematoma/abscess was considered clinically. Routine blood tests and urine analysis were within normal limits except leukocytosis (10,000/mm^3^) with neutrophilia. Ultrasonography (USG) of the right inguino-labial region revealed a mildly echogenic cystic swelling of size 2.80 cm × 1.35 cm below the inguinal ligament and medial to the femoral vessels without any intra-abdominal communication or ascites (Fig. 2). Color Doppler flow study failed to show any vascular component of this swelling. Ultrasonography also showed one right-sided inflamed superficial inguinal lymph node.

**Fig. 3:**
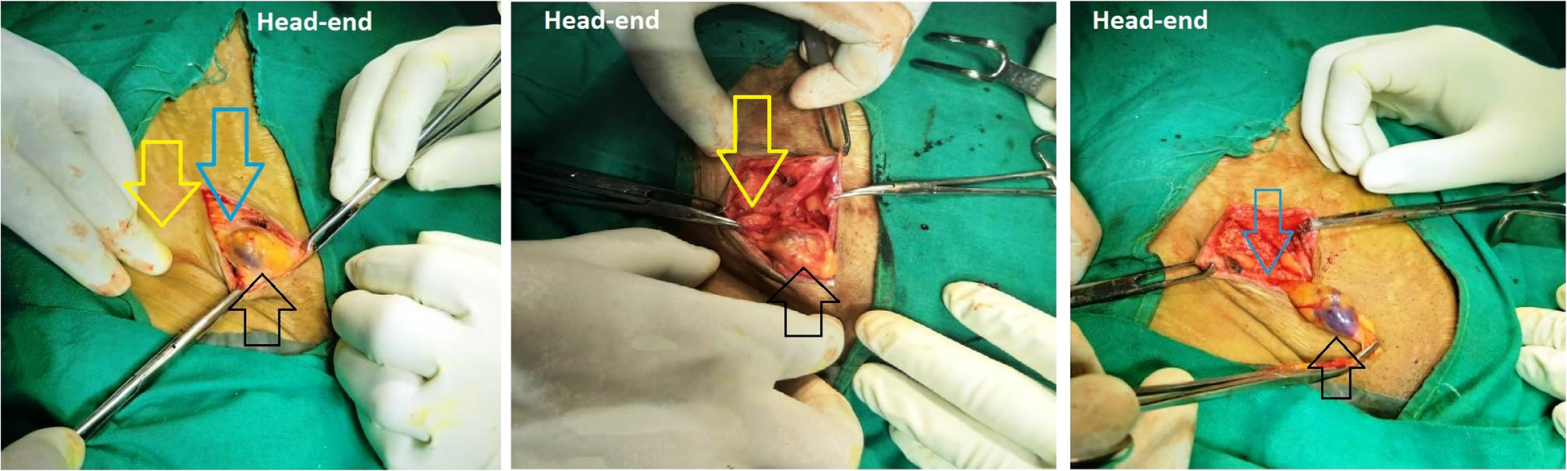
Intraoperative finding of the intact femorocele sac (thick-walled cyst containing amber-colored fluid) coming out of the femoral ring. Blue arrows, position of the inguinal ligament; black arrow, femorocele sac; yellow arrow, enlarged superficial inguinal lymph node

**Fig. 4:**
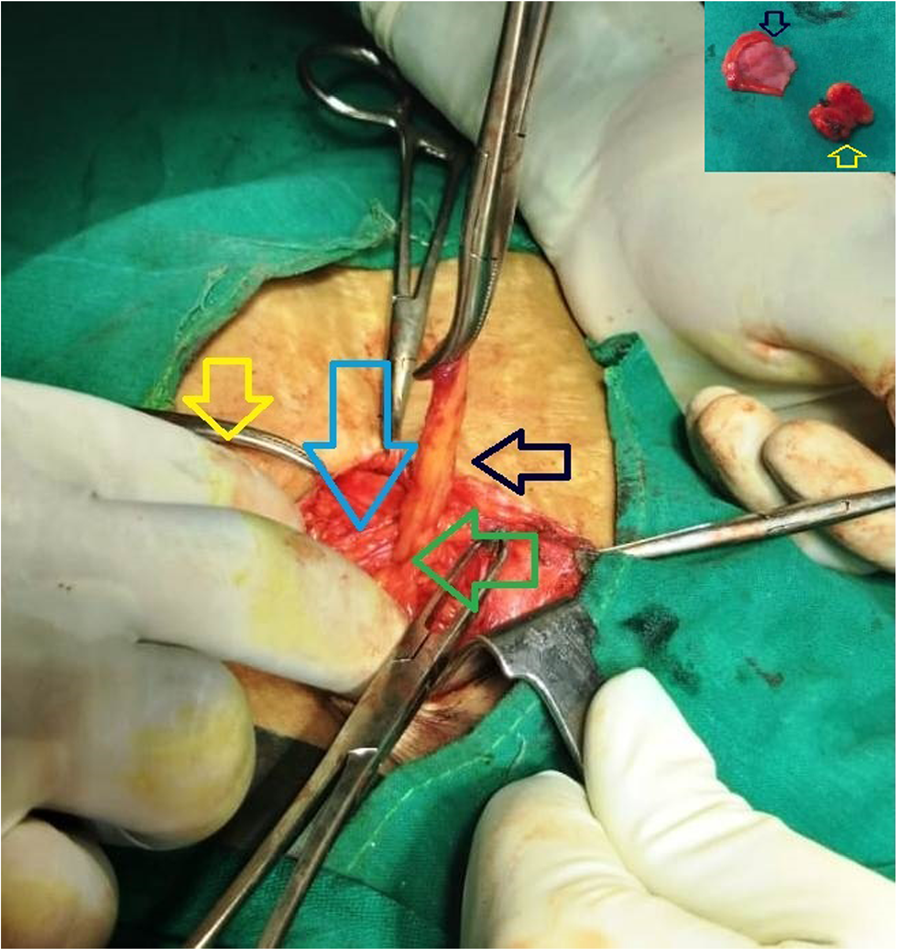
Femorocele sac (black arrow) coming out of the femoral ring (green arrow), and it is below the inguinal ligament (blue arrow). Yellow arrow, enlarged superficial inguinal lymph node. Inset: excised femorocele sac (black arrow) and harvested superficial inguinal lymph node (yellow arrow)

Exploration of right inguinal region under spinal anesthesia revealed thick-walled cyst (3 cm × 2 cm) containing approximately 5 ml of amber-colored fluid (Fig. 3). The possibility of herniated urinary bladder diverticulum was nullified as it failed to disappear after the urinary bladder catheterization. The cyst was found located medial to the femoral vessels, and its obliterated narrow neck-like continuation was seen below and behind the inguinal ligament. On opening the sac, the neck was found completely obliterated and there was no communication with the peritoneal cavity without any evidence of herniation. Therefore, the diagnosis of femorocele was confirmed intraoperatively. The obliterated neck region was transfixed, and the distal sac was excised (Fig. 4). Anatomical repair of femoral canal defect was done, without tension, by approximating the iliopectineal and inguinal ligament. The harvested right superficial inguinal lymph node (1.5 cm × 0.5 cm) along with excised femorocele sac was sent for histo-pathological examination. The cyst fluid was sent for microbiological culture and antibiotic sensitivity test.

**Fig. 5:**
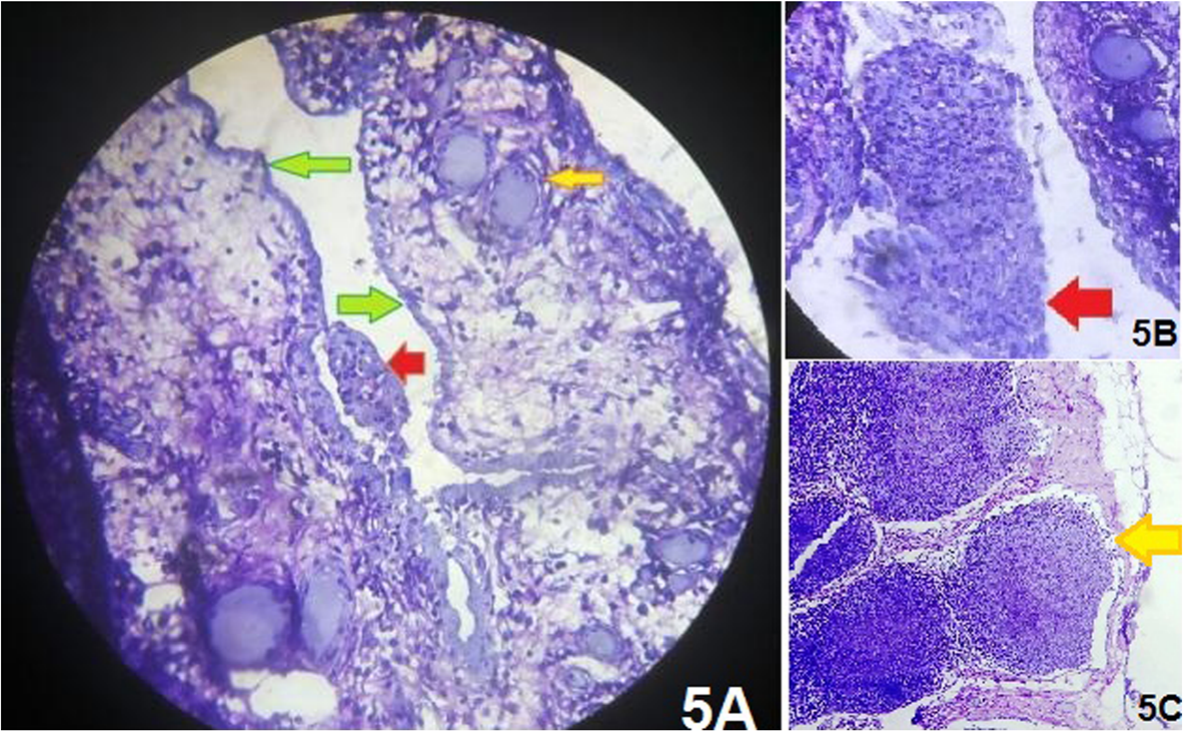
**a** Sections from the sac revealed fibro-fatty-collagenous tissue with mixed inflammatory cells. There were dilated lymphatics (yellow arrow) along with a flattened mesothelial lining cell layer (green arrows) suggestive of an infected peritoneal sac (femorocele). Red arrow shows mesothelial tissues (enlarged image in **b**) **c** Sections from the inguinal lymph node showed features of reactive hyperplasia (yellow arrow)

Sections from the sac revealed fibro-fatty-collagenous tissue with mixed inflammatory cells. There were dilated lymphatics along with a flattened mesothelial lining cell layer. This confirmed infected peritoneal sac (femorocele) (Fig. 5). No granuloma or malignancy was seen. Sections from the inguinal lymph node showed features of reactive hyperplasia. Culture of fluid from the sac revealed significant growth of *Escherichia coli*, which was sensitive to amikacin and imipenem.

Postoperative period in hospital was uneventful. She was put on anti-inflammatory drugs and antibiotics targeting uropathogens (i.e., amikacin, which later complied drug sensitivity report also). Oral feeding was started the next day. She became afebrile after 2 days, and early ambulation was encouraged. There was no surgical site infection. The patient was discharged in stable condition after 5 days. Four months after the operation, she is asymptomatic and has returned to her usual life without any clinical sign of recurrence. She was advised regular follow-up in our surgical outpatient department (OPD) to detect any recurrence by clinical examination and ultrasonography.

## Discussion

The hydrocele of the femoral hernia sac had been an extremely rare disease. In 1882, Marcy first suggested that cystic dilatation of a portion of the femoral hernia sac may form a complication. Sir Astley Cooper was the first scientist to describe the hydrocele of the femoral hernia sac, in 1884. When Desault brought a candle near the femoral hernia sac, he found it has a transilluminated swelling [4]. Erdman also found that a small tab of inflamed omentum with serous exudates simulating hydrocele in the femoral hernia sac [6]. Babcock agreed that cysts of the femoral canal are rare, irreducible, and impulseless. He opined that without exploration, it is difficult to differentiate it from an irreducible hernia. De Garmo wrote that the narrow femoral hernia neck may get obliterated completely after the use of truss and may form the hydrocele, which can be mistakenly diagnosed as an irreducible femoral hernia [4]. But nowhere, infection of femorocele fluid has been reported before.

The femoral canal is the medial-most compartment of the femoral sheath. The proximal opening of the femoral canal (also known as the femoral ring) is covered by a femoral septum which is condensed extraperitoneal fatty tissue. The canal normally contains loose areolar tissue, few lymphatics, and one deep inguinal lymph node called Cloquet’s node. The peritoneal sac may herniate through the femoral ring into the femoral canal, medial to the femoral vessels, to form femoral hernia. The presence of a potential or actual femoral hernia sac or embryonic peritoneal rest is the pre-requisite for femorocele formation. Different factors, like congenital, inflammatory, or traumatic occlusion of the communication with the peritoneal cavity, are responsible for the fluid collection in the femorocele sac like the hydrocele of the scrotum and spermatic cord in males and hydrocele of the canal of Nuck in females [2–4]. Femorocele fluid is amber-colored and sterile in nature, unless it gets infected. There is a presence of albumin and fibrinogen in the fluid.

Lean females in fourth to sixth decades of their life [7] are at increased risk of developing the femoral hernia and in rare consequences femorocele, owing to enlarged femoral ring. Clinically, femorocele presents as painless groin swelling. This never has been diagnosed preoperatively, but only after surgical exploration in all cases reported to date. Femorocele is of two types, viz.

1. Primary or true femorocele: Collection of fluid in the sac of femoral hernia either due to complete obliteration or omental plugging/adhesion at the narrow neck of the sac, with no evidence of ascites.
2. Secondary: Fluid collection in the sac of femoral hernia from the peritoneal cavity associated with the presence of ascites.

Commonly, irreducible or incarcerated femoral hernia, cyst of the canal of Nuck, subcutaneous lipoma, Bartholin’s cyst, lymph node abscess, arterial or venous aneurysms, and post-traumatic hematoma may mimic femorocele. Also, rare entities like cystic lymphangioma, ganglion cyst, and neuroblastoma metastasis [8–10] in the groin should be considered in differential diagnosis. Evaluation with USG and MRI may be used to reach the correct diagnosis [11]. Most studies have recommended primary tissue repair through a low approach, particularly if there is no tension or risk of wound infection. Excision of the sac after the transfixation of the neck and its reduction into peritoneal cavity and anatomical repair with obliteration of femoral ring by approximation of both ligaments with non-absorbable suture without tension should be the treatment of choice. The role of mesh in this disease is debatable. In elderly patients, with a larger defect and no wound infection risk, the mesh repair can be tried.

Infection of the femorocele fluid was not reported earlier. Like the hydrocele fluid, femorocele fluid may also get infected from adjoining epididymitis/infected urinary tract by uropathogens. In the present case, trauma might have led to hemorrhage and later infection of the femorocele fluid and adjacent superficial inguinal lymphadenopathy with fever. Excision of the the femorocele sac with the repair of the defect and use of sensitive antibiotics had cured the patient.

## Conclusions

The hydrocele of the femoral hernia sac is an extremely rare disease. When non-infected, it presents a painless inguinal soft cystic swelling, commonly in women of fourth to sixth decade. This was diagnosed intraoperatively in all cases reported to date. Excision of the sac after the transfixation of the neck and anatomical repair are the treatment of choice. In elderly patients, with larger defect, the mesh repair can be opted for.

The femorocele may also get infected by uropathogens and presents as pyrexia of unknown origin if one ignores its possibility to occur. When infected, it may also mimic incarcerated femoral hernia, inguinal abscess, or infected inguinal lymph node. Proper antibiotics should be used after a sensitivity test. Despite the rarity, surgeons dealing with femoral hernia should be aware of this clinical entity during the exploration of femoral hernia and suspected pathology involving the femoral hernia site.

## Data Availability

Presented within the manuscript, please contact the author for additional data requests.

## Abbreviations

BMI: Body mass index
IV: Intra-venous
MRI: Magnetic resonance imaging
USG: Ultrasonography
